# Prevalence and distribution of carbapenem‐resistant *Enterobacterales* in companion animals: A nationwide study in the United States using commercial laboratory data

**DOI:** 10.1111/jvim.17171

**Published:** 2024-08-17

**Authors:** Kurtis Sobkowich, Zvonimir Poljak, J. Scott Weese, Andy Plum, Donald Szlosek, Theresa M. Bernardo

**Affiliations:** ^1^ Department of Population Medicine University of Guelph, Ontario Veterinary College Guelph Ontario Canada; ^2^ Department of Epidemiology University of Guelph, Ontario Veterinary College Guelph Ontario Canada; ^3^ Ontario Veterinary College University of Guelph Guelph Ontario Canada; ^4^ IDEXX Laboratories Inc. Westbrook Maine USA

**Keywords:** antimicrobial resistance, epidemiology, microbiology, One Health, surveillance

## Abstract

**Background:**

Carbapenem‐resistant *Enterobacterales* (CRE) are a concern in both human and animal medicine globally. Despite extensive research in humans, limited data exist on CRE in companion animals, with a lack of nationwide prevalence estimates.

**Hypothesis/Objectives:**

To assess the occurrence and trends of CRE in cats and dogs across the United States by analyzing 4 years of commercial antimicrobial susceptibility testing (AST) data.

**Animals:**

Between 2019 and 2022, 477 426 ASTs were conducted on *Enterobacterales* isolates against imipenem. Isolates were derived from 379 598 dogs and 97 828 cats. Animal origin was not disclosed.

**Methods:**

In this retrospective study, antimicrobial susceptibility test data from IDEXX Laboratories were analyzed. Analysis included resistance estimations to imipenem stratified by sampling site, an assessment of resistance patterns over time and location, and the application of space‐time cluster analysis to identify potential outbreaks. Antibiograms were produced for carbapenem‐resistant isolates.

**Results:**

Susceptibility to imipenem was high, at 98.86%. Temporal analysis indicated stability in susceptibility, with an unexplained reduction in susceptible isolates in June 2019. Spatial analysis identified 2 high‐risk clusters along the Western Coast (relative risk [RR]: 23.26; *P* < .001) and in Texas (RR: 10.72; *P* < .001) in that month. Three other clusters were found, in Missouri (RR: 39.55; *P* = .038), Florida (RR: 4.53; *P* < .001), and New York (RR: 9.20; *P* < .001).

**Conclusions and Clinical Importance:**

CRE are present at a low prevalence in dogs and cats across the United States. Variations in prevalence across patient‐level and environmental factors highlight the need for tailored stewardship programs.

**Abbreviations**
AMR: antimicrobial resistance
AST: antimicrobial susceptibility testing
CLSI: Clinical and Laboratory Standards Institute
CRE: carbapenem‐resistant *Enterobacterales*
ESBL: extended‐spectrum beta‐lactamases
I: intermediate
LOESS: locally estimated scatterplot smoothing
MALDI‐TOF: matrix‐assisted laser desorption/ionization ‐ time of flight
MIC: minimum inhibitory concentration
R: resistant
RR: relative risk
S: susceptible
SST: skin and soft‐tissue
STL: season and trend decomposition using LOESS

## 1 INTRODUCTION

Carbapenems—a class of potent beta‐lactam antibiotics—are typically reserved for difficult to treat infections because of their efficacy against a range of bacteria, including those resistant (R) to other antimicrobials. Their resilience to extended‐spectrum beta‐lactamases (ESBL) enhances their effectiveness against ESBL‐producing *Enterobacterales*. The World Health Organization classifies carbapenems as critically important for both human and veterinary medicine.^1^ Therefore, resistance to these drugs is a global and multispecies health concern. Carbapenem‐resistant *Enterobacterales* (CRE) are of particular concern, as this order of bacteria is responsible for numerous common community and hospital acquired infections.

Carbapenem‐resistant *Enterobacterales* were first reported in the United States in 2001; a novel strain of R *Klebsiella pneumoniae*.^2^ Since then, CRE have become endemic in humans across the United States, albeit still at a relatively low incidence of 0.3 to 2.93 infections per 100 000 person‐years.^3^, ^4^ Evidence for zoonotic transmission of CRE between humans and animals remains limited; although, several studies highlight genotypic resemblances in R strains isolated from both sources.^5^, ^6^, ^7^, ^8^ Assuming that zoonotic transmission is possible, cats and dogs could serve as a frequent source of exposure, given the close contact they share with humans. Likewise, CRE could also be a zooanthroponotic threat for companion animals from humans.^9^

Data on the occurrence of CRE infections in companion animals, namely cats and dogs, are limited, and estimates are often based on low sample sizes or select regions. Limited formal surveillance programs are in place for the detection of CRE outbreaks in cats and dogs. Information obtained from clinical specimens sent to commercial diagnostic labs for culture and susceptibility testing can serve as a valuable source of antimicrobial resistance data at the population level.^10^ This approach benefits from a large sample size and uniform testing conditions across a wide geographic area. Although these data might lean toward representing more severe infections, as clients and veterinarians might choose not to culture and test because of financial or time constraints,^11^ they offer a distinct viewpoint on patterns at the population level across different locations and periods.

Using nationwide commercial antimicrobial susceptibility data from cat and dog isolates across the United States, this study aims to provide a high‐level epidemiological overview of CRE in companion animals. Spatial, and temporal trends in CRE prevalence are presented as well as susceptibility test results for over 30 bacterial species against carbapenems. Furthermore, this research provides antibiograms for alternative classes of antimicrobials, against isolates exhibiting resistance to carbapenems.

## 2 MATERIALS AND METHODS

Data for this study were obtained from 4 consecutive years (2019‐2022) of commercial antimicrobial susceptibility testing (AST) of bacterial isolates from cats and dogs submitted from across the United States to IDEXX Laboratories. Samples were submitted from both primary care clinics and tertiary animal hospitals; however, no indication was provided to differentiate the source of each sample. All culture and susceptibility tests were conducted by standardized methods, under standardized conditions, across IDEXX microbiology laboratories throughout the United States. The recorded data for each isolate included a deidentified animal identification number, date of sample collection (month/year), state and county of sample collection, infection site, bacteria isolated, and susceptibility results interpreted as susceptible (S), intermediate (I), or R. Minimum inhibitory concentration (MIC) data were not available in these data. Susceptibility interpretations were made before data handover and were based on site and isolate‐specific MIC breakpoints provided by the Clinical and Laboratory Standards Institute (CLSI) in the 4th edition of “*Performance Standards for Antimicrobial Disk and Dilution Susceptibility Tests for Bacteria Isolated from Animals*.”^12^ When veterinary‐specific breakpoints were unavailable, an appropriate human breakpoint was substituted.^13^

Isolates identified as *Enterobacterales* were eligible for inclusion in this cross‐sectional study. Bacterial identification and susceptibility testing were performed by automated means using VITEK2 (bioMérieux).^14^ In instances where the automated procedure was not available, bacterial identification was conducted via matrix assisted laser desorption/ionization ‐ time of flight mass spectrometry (MALDI‐TOF),^15^ and susceptibility testing was performed by the Kirby‐Bauer disk diffusion method.^16^ All isolates included were subjected to testing against imipenem, serving as an indicator for susceptibility to the carbapenem class of antimicrobials.

Records from the same animal, with the same isolated bacteria, the same MIC interpretation, and within 3 months of an earlier test were assumed to be results of duplicate testing and were removed from the dataset before analysis. The decision to use a 90‐day timeframe was based on findings that the majority of CRE infections in companion animals are resolved within this period.^17^ Because of the absence of clinical details in this study, isolates from different infection sites within 90 days were not assumed to be related, and all were retained for analysis.

Dog and cat animal populations were estimated per county by extrapolating human census population data from the United States Department of Agriculture (USDA)^18^ and converting to the number of households using an average of 2.6 people per household.^19^ Pet ownership statistics from the American Veterinary Medical Association were used to estimate the number of cats and dogs in each county, based on an estimate of 1.6 dogs and 1.8 cats per household.^20^ Counts of CRE isolates were divided by the estimated companion animal population to standardize the data when making geographic comparisons.

To infer patterns over time, total counts of observed S, I, and R interpretations were tallied by month and plotted on a time series graph. Susceptibility interpretations over time were presented as percentages of the total observations. A smoothed LOESS (locally estimated scatterplot smoothing) line and 95% confidence interval derived via t‐based approximation accompanied the data to assist with interpretation of the trend.

Space‐time cluster analysis was performed by the SatScan software,^21^ and the spatial scan statistic method.^22^ Spatial scanning was conducted to identify clusters of elevated resistance rates. A circular scanning window approach was employed, using a Poisson model with the estimated companion animal population per county as the denominator. Monte Carlo simulations were applied to assign a level of statistical significance to each cluster. The maximum cluster size was set to 50% of the population and 50% of the time interval. Relative risks were computed to evaluate the risk of observing a CRE infection within identified clusters compared to the risk in the population outside of these clusters. The spatial scan test was conducted only for the contiguous United States.

In accordance with the CLSI guidelines on “*Analysis and Presentation of Cumulative Antimicrobial Susceptibility Test Data (5th edition)*,” prevalence/resistance estimates were only calculated for bacterial species represented by a minimum sample size of 30.^23^ Any mention of nonsusceptibility henceforth refers to samples observed to possess a test interpretation of R or intermediately R. Where applicable, 95% confidence intervals are presented in square brackets.

## 3 RESULTS

After data cleaning, imipenem susceptibility data were available for 477 426 *Enterobacterales* isolates submitted from 2019 to 2022 from 424 018 unique animals: 79.5% (379 598) from dogs and 20.5% (97 828) from cats. The overall observed susceptibility to imipenem among *Enterobacterales* isolates was 98.86%, with 0.76% classified as I and 0.38% as R. The susceptibility rates for each year, were as follows: 98.64% (103 779/105 206) in 2019, 98.9% (116 051/117 346) in 2020, 98.96% (127 073/128 413) in 2021, and 98.89% (125 058/126 461) in 2022. The susceptibility of *Enterobacterales* to imipenem isolated from dogs was slightly lower at 98.7%, compared to 99.4% for those isolated from cats.

*Enterobacterales* were predominantly isolated from urine (68.5%, n = 326 827), and skin and soft‐tissue (SST) samples (13.7%, n = 65 322). Across all sites, susceptibility to imipenem was high, at greater than 97%. However, isolates from musculoskeletal, respiratory, and procedural sites (ie, incisions or implants) saw the lowest susceptibility to imipenem; 97.43% [96.16%, 98.37%], 97.75% [97.25%, 98.19%], and 97.65% [97.04%, 98.16%], respectively. Gastrointestinal and urinary samples showed the highest proportion of S isolates, at 99.24% [99.06%, 99.40%] and 99.06% [99.03%, 99.09%], respectively. Susceptibility to imipenem across all infections sites ranged from 97.43% to 99.24%, indicating that while some sites exhibit a marginal tendency toward lower susceptibility, the results were largely consistent across all infection sites. A complete breakdown of susceptibility interpretations by sampling site is provided in Supplementary Material 1.

The majority of *Enterobacterales* that were isolated belonged to the family Enterobacteriaceae (96.9%, n = 462 658), with *Escherichia* spp., *Klebsiella* spp., and *Enterobacter* spp., making up most observations. Enterobacteriaceae were interpreted as S to imipenem in 98.9% of tests. *Escherichia* ssp., *Klebsiella* spp., and *Enterobacter* spp. were observed to be S to imipenem in 99.6%, 93.5%, and 95.0% of tests, respectively. The family with the lowest susceptibility to imipenem was *Morganellaceae*, with 61.8% of isolates found to be S. This determination was based on a relatively limited sample size (n = 272). Some level of intrinsic resistance to imipenem has been document and is expected for certain genera of the *Morganellaceae* family. Most *Morganellaceae* isolates were *Proteus* species (84.9%, n = 231), which were S in 62.8% of tests. A complete breakdown of MIC interpretations by bacterial family and genus is presented in Table 1.

**TABLE 1:** Breakdown of imipenem susceptibility interpretations by taxonomic family and genus.

	Interpretation of MIC value						
	Susceptible		Intermediate		Resistant		
	Obs.	(%)	Obs.	(%)	Obs.	(%)	n
Enterobacteriaceae	4,57 394	98.9%	3596	0.8%	1668	0.4%	462 658
*Buttiauxella* spp.	104	99.0%	0	0.0%	1	1.0%	105
*Cedecea* spp.	–		–		–		27
*Citrobacter* spp.	6439	98.7%	53	0.8%	32	0.5%	6524
*Cronobacter* spp.	176	98.9%			2	1.1%	178
*Enterobacter* spp.	22 225	95.0%	758	3.2%	404	1.7%	23 387
*Escherichia* spp.	3,85 743	99.6%	517	0.1%	885	0.2%	387 145
*Klebsiella* spp.	36 804	93.5%	2241	5.7%	328	0.8%	39 373
*Kluyvera* spp.	151	98.1%	1	0.6%	2	1.3%	154
*Kosakonia* spp.	–		–		–		1
*Leclercia* spp.	2953	99.7%	4	0.1%	5	0.2%	2962
*Lelliottia* spp.	697	99.4%	2	0.3%	2	0.3%	701
*Plesiomonas* spp.	76	98.7%	0	0.0%	1	1.3%	77
*Pluralibacter* spp.	759	99.3%	2	0.3%	3	0.4%	764
*Pseudescherichia* spp.	–		–		–		1
*Raoultella* spp.	489	97.2%	13	2.6%	1	0.2%	503
*Salmonella* spp.	733	99.9%	0	0.0%	1	0.1%	734
*Shigella* spp.	–		–				4
*Yokenella* spp.	–		–		–		18
Erwiniaceae	12 808	99.9%	1	0.0%	12	0.1%	12 821
*Erwinia* spp.	118	100.0%	–		–		118
*Pantoea* spp.	12 690	99.9%	1	0.0%	12	0.1%	12 703
Hafniaceae	378	99.5%	0	0.0%	2	0.5%	380
*Edwardsiella* spp.							22
*Hafnia* spp.	356	99.4%			2	0.6%	358
Morganellaceae	168	61.8%	27	9.9%	77	28.3%	272
*Moellerella* spp.	–		–		–		3
*Morganella* spp.	–				–		25
*Proteus* spp.	145	62.8%	26	11.3%	60	26.0%	231
*Providencia* spp.	–		–		–		13
Pectobacteriaceae	–		–		–		1
*Brenneria* spp.							1
Yersiniaceae	1212	93.7%	27	2.1%	55	4.3%	1294
*Ewingella* spp.	335	99.7%	1	0.3%	0	0.0%	336
*Rahnella* spp.	43	100.0%	0	0.0%	0	0.0%	43
*Rouxiella* spp.	–		–		–		9
*Serratia* spp.	748	90.3%	26	3.1%	54	6.5%	828
*Yersinia* spp.	77	98.7%	–		1	1.3%	78

*Note*: Counts censored if total less than 30 observations. Discrepancies in column totals may exist because of censoring.

**FIGURE 1:**
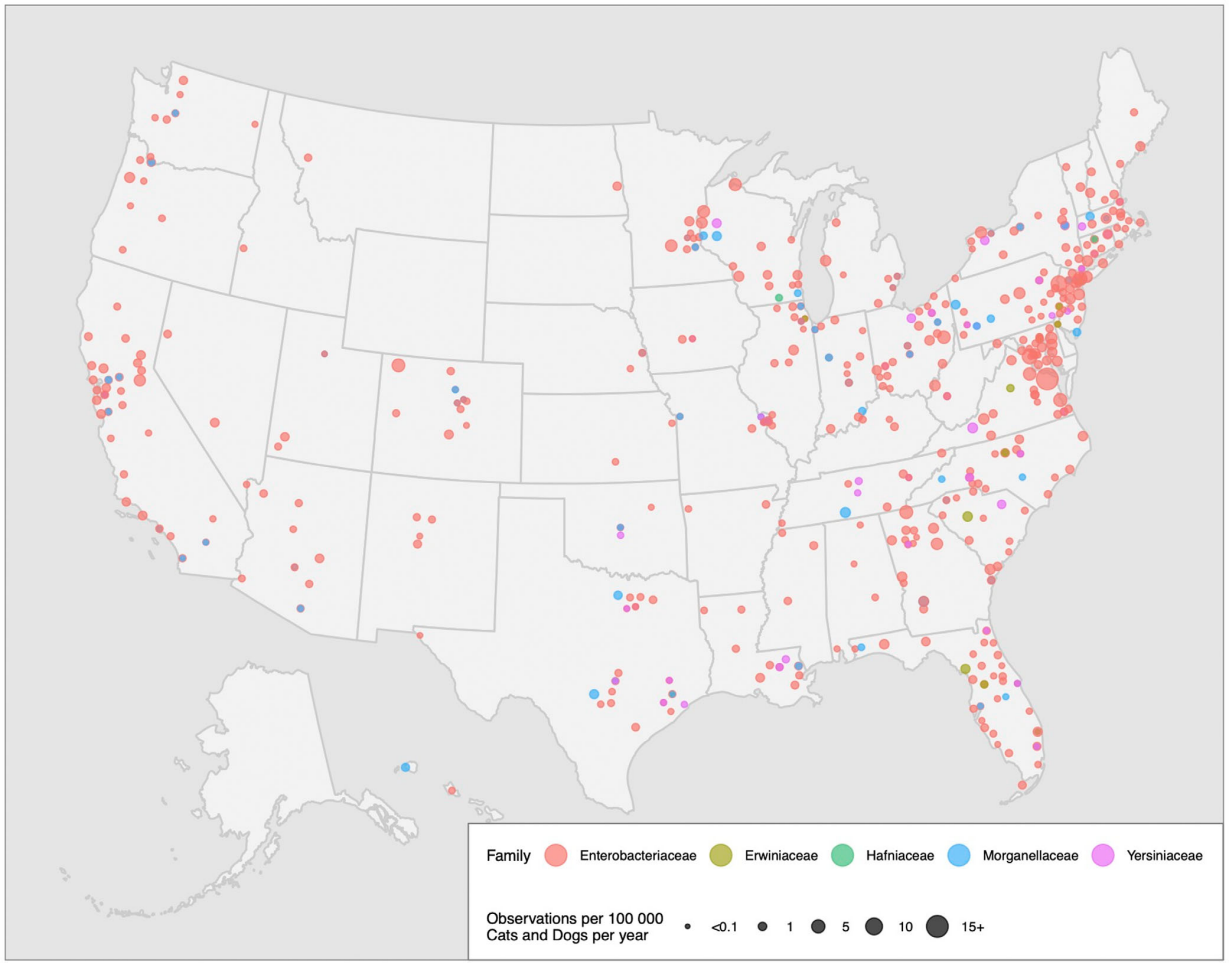
Observed CRE across the United States of America between 2019 and 2022 per 100 000 cats and dogs per year.

A map of the observations of imipenem R *Enterobacterales* between 2019 and 2022, stratified by taxonomic family and standardized by estimated companion animal population is presented in Figure 1. Visual assessment of this dot map indicates a large grouping of observations on the eastern coast, particularly in the north, and mainly made up of observations of Enterobacteriaceae. A tabular breakdown of susceptibility interpretations by state is presented in Supplementary Material 2.

**FIGURE 2:**
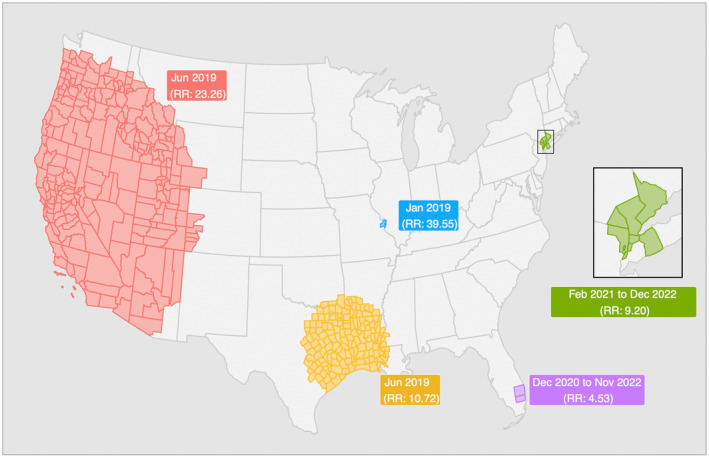
Identified space‐time clusters of CRE in companion animals across the contiguous United States of America between 2019 and 2022.

Five high‐risk clusters of CRE were identified through space‐time cluster analysis (Figure 2). Two clusters were found to exist for a single month in June 2019: 1 encompassing multiple west‐coast states (relative risk [RR]: 23.26; *P* < .001), and the other situated in the south‐central region of the country centered near Dallas, Texas (RR: 10.72; *P* < .001). A third cluster was identified for a single month in January 2019 near St. Louis, Missouri (RR: 39.55; *P* = .038). Two, more temporally stable, clusters were also identified: 1 in Southern Florida (RR: 4.53; *P* < .001), persisting from December 2020 to November 2022, and another in the state of New York near New York City (RR: 9.20; *P* < .001), spanning from February 2021 to the conclusion of the study period in December 2022.

**FIGURE 3:**
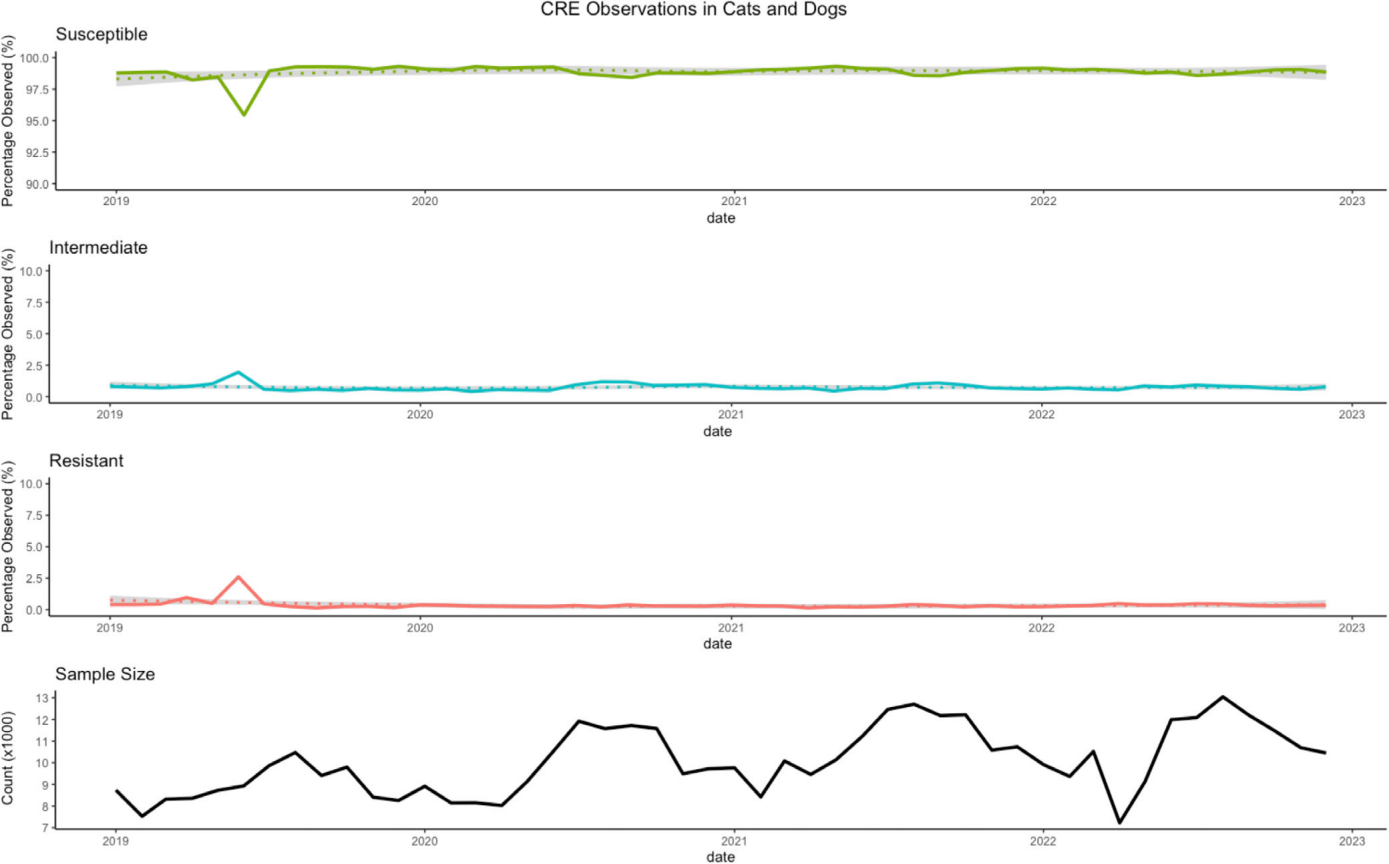
Time series plot of antimicrobial sensitivity test results for *Enterobacterales* tested against imipenem.

Throughout the study period, the percentage of S isolates per month remained relatively stable, exhibiting some indication of seasonality, with a declining proportion of susceptibility observed during the late summer (Figure 3). Seasonal‐Trend decomposition using LOESS (STL decomposition) provided marginal evidence toward the presence of a repeating annual pattern in the proportion of CRE observations, with a lower proportion of susceptibility in the late summer months. An abrupt decrease in the proportion of S isolates was seen in the month of June 2019, coinciding with the aforementioned high‐risk clusters in the western states and near Texas. Further investigation into this spike in resistance, revealed that most of these observations were from Enterobacteriaceae isolates, which saw a 140.16% increase in resistance from May to June 2019, before reverting to previous levels in July. On average, 107.6 nonsusceptible isolates were observed each month, excluding June 2019. In June 2019, there were 407 nonsusceptible isolates observed, exceeding the monthly average by 300. Two genera accounted for 295 of the isolates in this surplus and displayed substantial deviations from their respective monthly averages of nonsusceptible isolates observed. *Escherichia* exhibited an 1157.2% increase, reporting 291 nonsusceptible isolates in June 2019, compared to its monthly average of approximately 23. Similarly, *Enterobacter* demonstrated a notable 115.8% increase, with 50 nonsusceptible isolates in June 2019, surpassing its monthly average of approximately 23. These findings were not determined to be the result of repeat testing or duplicated animal records. A plot of the number of R isolates per month by common genera is presented in Supplementary Material 3.

**FIGURE 4:**
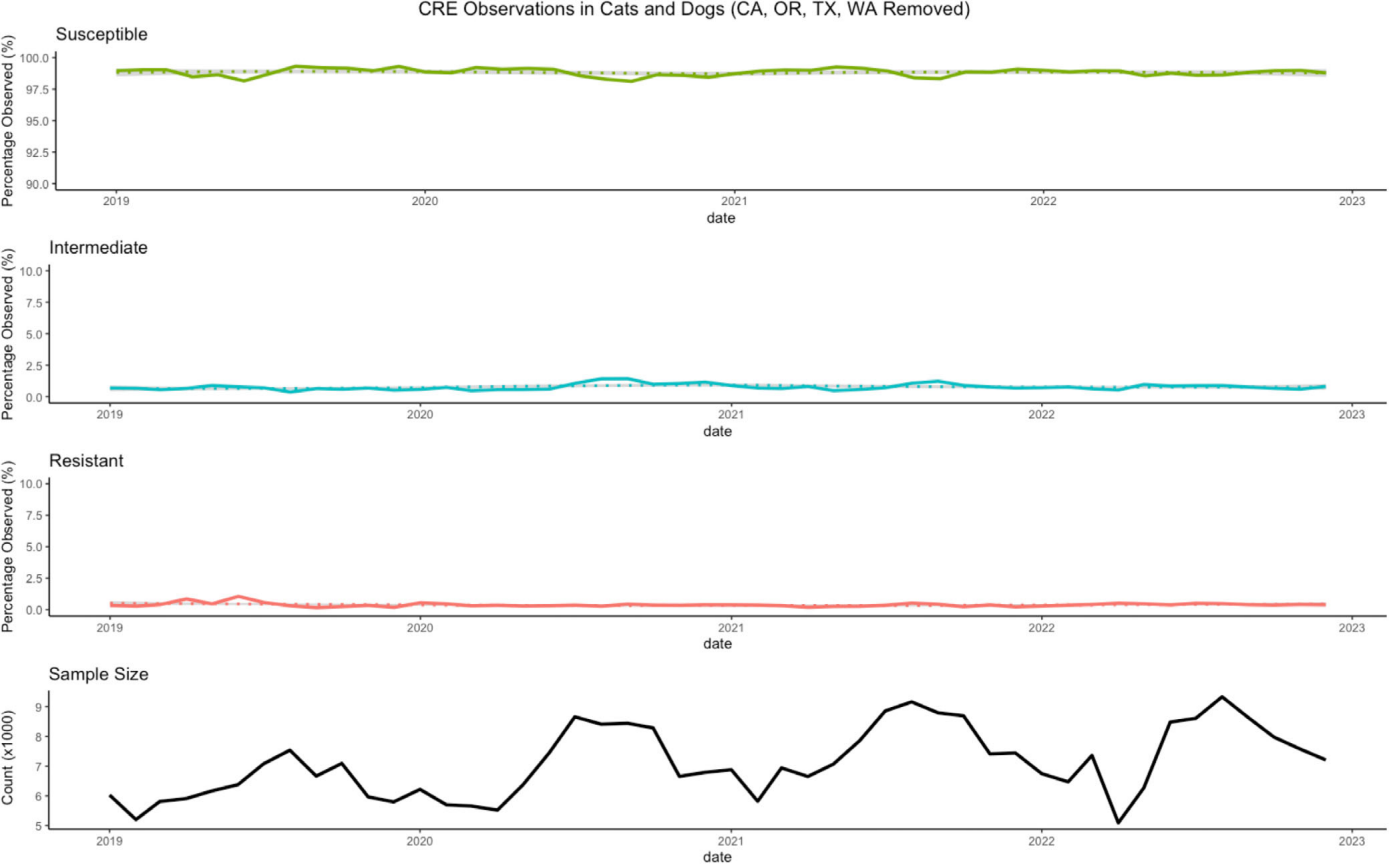
Time series plot of antimicrobial sensitivity test results for *Enterobacterales* tested against imipenem with California, Oregon, Texas, and Washington states removed.

Figure 4 presents a time series plot equivalent to that of Figure 3, displaying the observed *Enterobacterales* isolates tested against imipenem, with data from California, Oregon, Texas, and Washington states excluded (related to the June 2019 cluster). The exclusion of data points from these 4 states significantly reduces the pronounced lower susceptibility observed in June 2019.

Two antibiograms were developed for carbapenem‐resistant isolates, 1 stratified by common sampling sites (Table 2), and a second stratified by common bacterial isolates (Table 3). Among the drug classes tested, aminoglycosides demonstrated the highest overall effectiveness across all sample sites, with 89.4% [87.1%, 91.4%] of the isolates from SST, 88.7% [87.3%, 90.1%] from urinary tract, and 85.6% [83.5%, 87.5%] from all other sites showing susceptibility. Tetracyclines showed the lowest efficacy (ignoring beta‐lactams), for SST (18.0% [14.5%, 22.0%]) and other isolates (23.4% [20.0%, 27.2%]), yet remained moderately effective against urinary isolates (49.1% [46.0%, 52.3%]). Carbapenem‐resistant *E. coli* appeared to remain largely S to Fosfomycin (phosphonic; 97.5% [94.3%, 99.2%]), aminoglycosides (86.6% [85.0%, 88.1%]), and polymyxins (83.1% [71.0%, 91.6%]). Likewise, carbapenem‐resistant *Enterobacter* spp., and *Klebsiella* spp. isolates were observed to have moderately high susceptibility to aminoglycosides, at 89.6% [87.4%, 91.6%] and 85.8% [83.1%, 88.2%], respectively.

**TABLE 2:** Antibiogram of carbapenem‐resistant isolates stratified by sampling site.

	Skin and soft‐tissue						Urine						Other					
	Susceptible		Intermediate		Resistant		Susceptible		Intermediate		Resistant		Susceptible		Intermediate		Resistant	
	Obs.	(%)	Obs.	(%)	Obs.	(%)	Obs.	(%)	Obs.	(%)	Obs.	(%)	Obs.	(%)	Obs.	(%)	Obs.	(%)
Aminoglycosides	749	89.4%	8	1.0%	81	9.7%	1765	88.7%	32	1.6%	192	9.7%	1024	85.6%	13	1.1%	159	13.3%
Amphenicols	202	47.9%	131	31.0%	89	21.1%	449	45.0%	336	33.7%	212	21.3%	281	46.1%	154	25.3%	174	28.6%
Beta‐lactam	28	6.7%	5	1.2%	382	92.0%	172	17.3%	13	1.3%	809	81.4%	47	8.8%	11	2.1%	477	89.2%
First Gen Cephalosporin	20	4.8%	1	0.2%	397	95.0%	168	16.9%	21	2.1%	805	81.0%	48	8.9%	11	2.0%	483	89.1%
Third Gen Cephalosporin	611	31.1%	48	2.4%	1303	66.4%	1390	29.3%	60	1.3%	3301	69.5%	669	27.3%	30	1.2%	1756	71.5%
Nitrofurantoin	–		–		–		451	46.1%	207	21.2%	320	32.7%	12	42.9%	6	21.4%	10	35.7%
Penicillins	2	0.9%	8	3.4%	223	95.7%	68	8.5%	103	12.8%	633	78.7%	17	4.4%	19	4.9%	349	90.6%
Phosphonics	–		–		–		198	94.7%	5	2.4%	6	2.9%	–		–		–	
Polymyxins	–		–		–		–		–		–		61	58.7%	0	0.0%	43	41.3%
Quinolones	734	58.0%	27	2.1%	504	39.8%	1731	58.0%	59	2.0%	1197	40.1%	775	46.7%	46	2.8%	838	50.5%
Tetracyclines	78	18.0%	2	0.5%	353	81.5%	491	49.1%	22	2.2%	486	48.6%	128	23.4%	3	0.5%	415	76.0%
Trimethoprim	278	67.8%	0	0.0%	132	32.2%	713	72.2%	3	0.3%	271	27.5%	348	65.0%	1	0.2%	186	34.8%

*Note*: Counts censored if total less than 30 observations.

**TABLE 3:** Antibiogram of carbapenem‐resistant isolates stratified by bacterial species.

	*E. coli*						*Enterobacter* spp.						*Klebsiella* spp.					
	Susceptible		Intermediate		Resistant		Susceptible		Intermediate		Resistant		Susceptible		Intermediate		Resistant	
	Obs.	(%)	Obs.	(%)	Obs.	(%)	Obs.	(%)	Obs.	(%)	Obs.	(%)	Obs.	(%)	Obs.	(%)	Obs.	(%)
Aminoglycosides	1683	86.6%	39	2.0%	221	11.4%	794	89.6%	8	0.9%	84	9.5%	634	85.8%	4	0.5%	101	13.7%
Amphenicols	409	41.8%	392	40.1%	177	18.1%	182	41.6%	136	31.1%	119	27.2%	173	45.5%	61	16.1%	146	38.4%
Beta‐lactam	186	19.7%	15	1.6%	742	78.7%	0	0.0%	0	0.0%	433	100.0%	13	3.6%	5	1.4%	342	95.0%
First Gen Cephalosporin	188	19.9%	23	2.4%	733	77.6%	0	0.0%	1	0.2%	432	99.8%	13	3.6%	0	0.0%	348	96.4%
Third Gen Cephalosporin	1372	30.4%	16	0.4%	3118	69.2%	363	18.2%	81	4.1%	1553	77.8%	270	16.1%	11	0.7%	1395	83.2%
Nitrofurantoin	386	63.4%	95	15.6%	128	21.0%	56	31.1%	82	45.6%	42	23.3%	16	9.4%	36	21.1%	119	69.6%
Penicillins	61	6.5%	120	12.7%	762	80.8%	–		–		–		1	0.3%	8	2.2%	351	97.5%
Phosphonics	195	97.5%	2	1.0%	3	1.5%	–		–		–		–		–		–	
Polymyxins	49	83.1%	0	0.0%	10	16.9%	–		–		–		–		–		–	
Quinolones	1500	52.6%	57	2.0%	1294	45.4%	743	56.8%	28	2.1%	537	41.1%	399	36.6%	23	2.1%	667	61.2%
Tetracyclines	406	42.7%	2	0.2%	542	57.1%	151	34.2%	23	5.2%	268	60.6%	95	26.2%	1	0.3%	266	73.5%
Trimethoprim	609	65.4%	4	0.4%	318	34.2%	270	62.9%	0	0.0%	159	37.1%	272	76.2%	0	0.0%	85	23.8%

*Note*: Counts censored if total less than 30 observations.

## 4 DISCUSSION

This study aimed to assess the epidemiology of CRE in companion animals across the United States. Using 4 years of AST data from a national laboratory, the overall prevalence of CRE was estimated to be low, with 98.9% of observed *Enterobacterales* isolates being S to imipenem. Susceptibility was found to be fairly uniform across all sites, with only a minor tendency toward decreased susceptibility in musculoskeletal, respiratory, and procedural sites. Susceptibility across all sites ranged from 97.43% to 99.24%, with a spread of up to 1.8%. These findings are not substantial enough to definitively indicate any clinically relevant variation in susceptibility among sampling sites. The overall high degree of susceptibility is consistent with previous attempts to quantify the burden of CRE in both human^4^ and animal species.^24^, ^25^

Among Enterobacteriaceae, which composed the majority of isolates tested, *Klebsiella* spp. were least S (93.5% S) to imipenem. However, the majority of the nonsusceptible *Klebsiella* isolates fell only into the I category (5.7%) as opposed to be classified as completely R (0.8%). The earliest reported case of CRE in the United States was an isolate of carbapenem‐resistant *K. pneumoniae*, in 2001,^2^ and since then, *K. pneumoniae* carbapenemases have become the most common carbapenemase in the United States.^26^ Therefore, a reduced level of susceptibility in *Klebsiella* isolates is expected. When considering strictly R test results and ignoring *Morganellaceae* (further elaborated upon subsequently), *Enterobacter* spp. was found to be most R among the Enterobacteriaceae family (1.7% of isolates R), and *Serratia* spp. was most R overall in all *Enterobacterales* (6.5% of isolates R). Mechanisms of resistance against imipenem and other carbapenems in *Enterobacter* spp. and *Serratia* spp. have been previously described^27^, ^28^ but the overall prevalence of resistance is undocumented at a national level.

The bacterial family *Morganellaceae* were least S to imipenem (61.8% susceptibility). Several genera within the *Morganellaceae* family, specifically *Morganella*, *Proteus*, and *Providencia*, are known to exhibit some degree of intrinsic resistance to imipenem through mechanisms other than carbapenemases.^13^ Consequently, guidelines have been established to suggest that these genera undergo testing with a carbapenem other than imipenem before being deemed carbapenem‐resistant.^29^ However, this dataset did not provide susceptibility results for alternative carbapenems. This introduces a limitation in generalizing the susceptibility of these *Morganellaceae* to the broader carbapenem class. Although the *Morganellaceae* family exhibited notably reduced susceptibility relative to other families, they were not observed to show complete resistance, and the majority of *Proteus* isolates were imipenem S at 62.8%. *Morganellaceae* isolates made up only about 0.05% of the total isolates and did not substantially influence on any aggregated analysis. Importantly, none of the spatial clusters identified could be attributed to *Morganellaceae* outbreaks.

Temporal assessment revealed relative stability in the proportion of S tests over the 4 years studied, indicating that the issue of CRE in companion animals is not shifting, at least not at a rapid enough rate to be observed in only 4 years' time. Some degree of repeating seasonality appeared to be present, with a lower proportion of S isolates observed in the late summer. Limited research exists describing the seasonality of CRE in animals, but these findings appear to be consistent with observations made in human CRE cases in South Korea,^30^ which hypothesized that the greater occurrence of CRE infections could be related to temperature and humidity mediating transmission. Minimum inhibitory concentration values for tested isolates were not available in this study, but it is possible that with this more nuanced information that more subtle trends over time might have been observed.

Making use of commercial AST data, this study was able to assess the nationwide burden of CRE with comparable testing conditions and sample populations, allowing for geographic patterns and trends to be discerned. Mapping CRE cases revealed that R isolates were present in a substantial portion of the United States, showing a tendency to occur more frequently in regions with higher dog and cat population densities, using human population as an approximator. The map produced in this study closely mirrors patterns observed in maps of human CRE cases in the United States,^31^ providing some evidence that human and companion animal infections are correlated. However, this correlation might be confounded by human population density, and in turn, companion animal population density.

Among the 5 high‐risk clusters that were identified, 2 stand out, as they spanned substantial areas of the country for just 1 month. In June 2019, there was a significant decrease in the percentage of tests indicating susceptibility to imipenem. Even though the proportion of samples showing susceptibility decreased, other factors such as the total number of isolates tested, the geographic spread of testing, the proportion of bacterial genera isolated, and the species from which samples were collected remained stable. About 300 more nonsusceptible isolates were observed this month than compared to the monthly average. Of these 300 observations, 295 could be attributed to *Escherichia*, and *Enterobacter* which saw substantial increases in the number of nonsusceptible isolates observed in June 2019, compared to their respective monthly averages. This cluster appears to have spanned a sizeable portion of the West‐coast states and the Dallas, Texas region. A lack of information regarding the clinical context of the sample or the specific testing laboratory makes it challenging to determine whether this cluster signifies a genuine CRE outbreak or if it might be related to a testing procedure anomaly. No similar occurrences were found in reports on CRE in companion animals, livestock, or humans. Whether this discovery represents a valid finding, a transcription error in data recording, or a testing error remains uncertain, but after a reasonable investigation, no additional irregularities could be identified. Regardless, this finding highlights the need for surveillance systems to be in place to detect such anomalies in the future. These systems can be instrumental in initiating public health alerts or facilitating laboratory quality control efforts.

The remaining clusters, observed in Florida, Missouri, and New York, are suspected to be a result of referral bias from large veterinary clinics. In the data used, the locations associated with each sample correspond to the submitting veterinary clinic's location, introducing a potential bias in aggregation. In other words, the location recorded is that of the veterinary clinic submitting the sample, and not necessarily the residence of the animal. Consequently, areas with a higher concentration of veterinary clinics or larger more specialized clinics might be overattributed. Several veterinary referral hospitals are situated within each of these clusters, including St. Louis (MO), New York City (NY), and Fort Lauderdale (FL). The combination of highly specialized caseloads and the broad geographic service range could account, at least in part, for these identified clusters. Prior research has indicated that the occurrence of CRE in animals treated at veterinary hospitals is notably higher compared to pets in the general community, with an odds ratio of 12.8.^32^ However, the data used in this study do not differentiate samples received from a primary or tertiary point of care center, and therefore no correlation could be drawn. For effective surveillance of CRE to occur in the future, AST data records should include information about the level of care center from which the isolate is being sent. In the case of tertiary hospital samples, it is also important that the home location of the infected animal be included, to avoid artificial clustering in the data.

The World Health Organization lists carbapenems as “critically important” for human medicine, meaning that they are one of a limited few antibiotics that can treat serious multidrug R infections.^1^ In veterinary medicine, carbapenems are generally used as off‐label treatments of last‐resort for infections where limited therapeutic alternatives are available and there is a high probability of survival with their administration.^33^ Hence, any degree of resistance observed should be viewed with concern, as it suggests the potential ineffectiveness of other antibiotic classes as well. This served as the motivation behind creating antibiograms tailored to carbapenem‐resistant Enterobacteriaceae (CRE) isolates, aiming to identify potential alternative treatment options for CRE infections. Unsurprisingly, beta‐lactams and their derivatives (cephalosporin and penicillin antibiotics) were found to be largely ineffective against carbapenem‐resistant isolates. While older antibiotics, namely aminoglycosides, phosphonics, and polymyxins, all showed relatively high levels of effectiveness against CRE infections, in line with previous recommendations for treating gram‐negative multidrug R infections.^34^ Although these results support previous recommendations, treatment decisions for CRE infections should still be guided by susceptibility testing whenever possible. This should be done in conjunction with patient‐specific considerations and adherence to antimicrobial stewardship guidelines.^35^

The utilization of commercial AST data comes with certain constraints. Typically, in the United States, the decision to perform culture and sensitivity testing for companion animals is made on a case‐by‐case basis, and this decision is reached through mutual agreement between the veterinarian and the client, often guided by client financial considerations and risk assessment. This approach could potentially lead to a bias favoring wealthier demographics and specific regions. Furthermore, because of the cost of testing, many clients might choose to forgo testing and instead opt for a broad spectrum first‐line prescription, only choosing to test if the initial treatment fails. Consequently, it is reasonable to assume that the data contained within these datasets are skewed toward animals with refractory or recurrent infections, often after failure of empirical treatment. This creates a potential bias toward higher prevalence of carbapenem resistance in the dataset. Nonetheless, considering the tendency of many CRE infections to not respond to initial treatments, it is plausible to suggest that the dataset still accurately encompasses a substantial portion of such cases, despite any initial attempts at treatment.

In conclusion, although carbapenem resistance appears to be present in only a small proportion of *Enterobacterales* infections of dogs and cats, instances of resistance are occurring throughout the United States. Carbapenem‐resistant Enterobacteriaceae are among the World Health Organization's critical list of emerging AMR threats,^36^ and therefore any level of observed resistance is of concern. Effective surveillance systems should be implemented to identify outbreaks of CRE in a timely manner, but this will require large amounts of representative data. Laboratory AST data is a promising source for country‐level surveillance, but several biases should be quantified and reduced, namely understanding veterinarian testing, and prescribing behaviors, and ensuring that testing is occurring in a representative sample of the general population of cats and dogs. Assuming that some degree of zoonotic/zooanthroponotic transmission is likely occurring,^8^, ^9^ an integrated One Health approach will be needed to monitor the status of CRE. The results of this study offer an update on the epidemiology of CRE in companion animals in the United States, which can be expanded upon in future years.

## CONFLICT OF INTEREST DECLARATION

Andy Plum and Donald Szlosek were employed by the IDEXX Laboratories, Inc. Theresa Bernardo holds the IDEXX Chair in Emerging Technologies and Preventive Healthcare. Kurtis Sobkowich was supported by the IDEXX Chair. No other authors declare a conflict of interest.

## OFF‐LABEL ANTIMICROBIAL DECLARATION

Authors declare no off‐label use of antimicrobials.

## INSTITUTIONAL ANIMAL CARE AND USE COMMITTEE (IACUC) OR OTHER APPROVAL DECLARATION

Authors declare no IACUC or other approval was needed.

## HUMAN ETHICS APPROVAL DECLARATION

Authors declare human ethics approval was not needed for this study.

## Supporting information


**Supplementary Material 1.** Breakdown of MIC interpretations from carbapenem‐tested *Enterobacterales* by infection site.

**Supplementary Material 2.** Breakdown of MIC interpretations from carbapenem‐tested *Enterobacterales* by infection site.

**Supplementary Material 3.** Breakdown of observed nonsusceptible *Enterobacterales* by genus between 2019 and 2022.
