# Supplementary figures and images for: Prevalence and distribution of carbapenem‐resistant Enterobacterales in companion animals: A nationwide study in the United States using commercial laboratory data
Source: J Vet Intern Med. 2024 Aug 17;38(5):2642–53. doi: 10.1111/jvim.17171 (PMC11423476; doi:10.1111/jvim.17171)

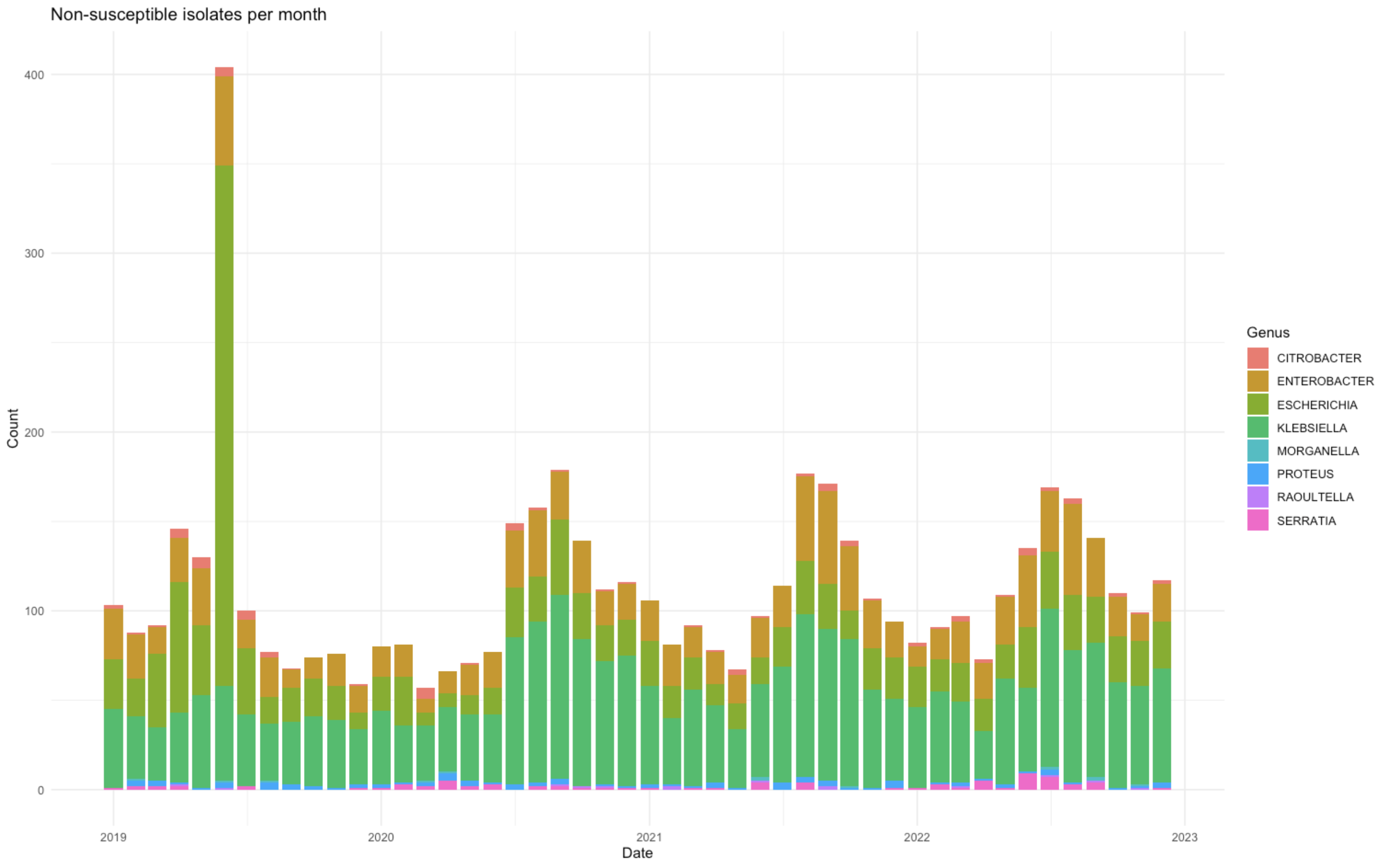

Supplement: Supplementary file 1 — Supplementary Material 1. Breakdown of MIC interpretations from carbapenem‐tested Enterobacterales by infection site. [file JVIM-38-2642-s002.png]
